# Ubiquitous Computing and Ambient Intelligence—UCAmI

**DOI:** 10.3390/s19184034

**Published:** 2019-09-19

**Authors:** Macarena Espinilla, Vladimir Villarreal, Ian McChesney

**Affiliations:** 1 Departament of Computer Science, University of Jaen, 23071 Jaén, Spain; 2 Technological University of Panama, Lassonde, West Avenue, David, CH, POB 6-2894, Panama; vladimir.villarreal@utp.ac.pa; 3 Pervasive Computing Research Group, School of Computing, Ulster University, Newtownabbey BT37 0QB, UK; ir.mcchesney@ulster.ac.uk

The Ubiquitous Computing (UC) idea envisioned by Weiser in 1991 [1] has recently evolved to a more general paradigm known as Ambient Intelligence (AmI) that represents a new generation of user-centred computing environments and systems. These solutions aim to find new ways to better integrate information technology into everyday life devices and activities.

AmI environments are integrated by several autonomous computational devices of modern life ranging from consumer electronics to mobile phones. Ideally, people in an AmI environment will not notice these devices, but will benefit from the services these solutions provide them. Such devices are aware of the people present in those environments by reacting to their gestures, actions, and context [2]. Recently the interest in AmI environments has grown considerably due to new challenges posed by society’s demand for highly innovative services, such as smart environments, Ambient Assisted Living (AAL), e-Health, Internet of Things, and intelligent systems, among others.

There are many challenges in this area of UC and AmI that need to be solved or improved. The special issue “Selected Papers from UCAmI 2018—The 12th International Conference on Ubiquitous Computing and Ambient Intelligence” of the Sensors journal seeks to explore new proposals in order to contribute to aspects such as: Design and implementation of wearable sensors and embedded technologies in ambient assisted living contexts, location in ambient intelligence or applications of ubiquitous computing and ambient intelligence.

For this purpose, of all the manuscripts received, only 11 original and high-quality manuscripts were selected for inclusion in this special issue. Each manuscript was reviewed by prestigious researchers in the same topics as the articles, and underwent up to three rounds of peer-review.

Each of the articles presents advances in research in UC and AmI applied to a range of interesting and relevant application domains. We hope that this special issue provides an inspirational collection of ideas, techniques, and methodologies for UC and AmI, which will continue to stimulate further research within this state-of-the-art domain.

In the first paper, “Assisting Visually Impaired People in the Public Transport System through RF-Communication and Embedded Systems” [3], a new approach is described to provide autonomous mobility in the public transport system to visually impaired persons by means of radio frequency communication and embedded systems. The main advantages of this new proposal are that it is low cost, user-friendly, modular, and requires no audio cues, which make it a good alternative to the current approaches.

In the context of road-based mass transit systems, the paper entitled “Bus Travel Time Prediction Model Based on Profile Similarity” [4] presents a short-term travel time prediction model for public transport buses. To estimate the travel time, the model takes into account historical behaviour, represented by medoids resulting from a clustering process based on the k-medoids technique, and the current travel time behaviour. The proposed method was applied in two real cases of public transport lines of different characteristics. The results showed that, in general, the average error made in the predictions is around 13% of the observed time travel values, a result similar to that obtained by the alternative artificial neural networks (ANN) method, although its variability is less than this alternative method.

In the paper entitled “Smart Management Consumption in Renewable Energy Fed Ecosystems” [5] a model of integration for the development of energy management facilities is proposed. The model is based on the use of Internet of Things (IoT) communication protocols, and artificial intelligence paradigms applied for classification, detection, prediction, and control of consumption and power generation systems. The artificial intelligence algorithms were deployed at two levels, the edge level and the fog level. The experimental results presented the benefits of the model: The ease of the design, installation, and operation, as well as optimized costs for the level of services developed.

The fourth paper, “Semi-Automated Data Labeling for Activity Recognition in Pervasive Healthcare”, is focused on semi-automatic labeling [6], proposing two approaches for semi-automated online data labeling performed by the individual executing the activity of interest. The first approach is based on the recognition of subtle finger gestures performed in response to a data-labeling query. The second approach focuses on labeling activities that have an auditory manifestation and uses a classifier to obtain an initial estimation of the activity, and a conversational agent to ask the participant for clarification or for additional data. Both approaches were evaluated in controlled experiments to assess their feasibility and their advantages and limitations. Results showed that while both studies had limitations, they achieved 80% to 90% precision.

In the context of indoor positioning systems (IPS), the paper entitled “Beacon-Related Parameters of Bluetooth Low Energy: Development of a Semi-Automatic System to Study Their Impact on Indoor Positioning Systems” [7] presents a semi-automatic data collection support system in a Bluetooth low energy (BLE) fingerprinting-based IPS. The aims of the proposed system are to streamline and shorten the data collection process, carry out impact studies by protocol, and channel on the static positioning accuracy related to configuration parameters of beacons such as transmission power, the advertising interval or geometric distribution.

The paper entitled “Protocol for Streaming Data from an RFID Sensor Network” [8] proposes a high read rate protocol, named sFSA, to stream data from an RFID sensor network. The proposed protocol increases the Sensor Read Rate (SRR), defined as the number of sensor data reads per second, compared to the standard. Additionally, this paper presents a prototype of an RFID sensor network to compare the proposed sFSA with the standard, increasing the SRR by more than five times on average. The experiment performed determined the possibilities of further improving the proposed sFSA working in real environments where the number of tags is unknown.

This special issue includes a “Systematic Literature Review of Food-Intake Monitoring in an Aging Population” [9]. In this review, the most important proposed technologies and techniques are analyzed in order to identify whether they can be applied in this context and if they can be used to improve the quality of life of this fragile collective. Three hundred and twenty-six papers were evaluated in which 29 proposals were completely analyzed, taking into account the characteristics and requirements of aging populations.

The paper entitled “DNS/DANE Collision-Based Distributed and Dynamic Authentication for Microservices in IoT” [10] uses a soft delegation schema, with a DNSSEC forwarder at the fog level, to propose a DNS-based dynamic authentication for a microservice architecture in IoT. The proposed fog DNS performs frequent updates to the DNS to cope with the demand of microservice creation, migration, and re-instantiation. The proposal uses a transferable, key exposure-free scheme of chameleon signatures, with a low computation cost for the fog forwarder and verifiers, which uses DNS-based Authentication of Named Entities to offer Transport Layer Security Authentication (TLSA) records, which can be used for establishing TLS secured sessions for microservices to interact with.

The ninth paper is entitled “Edge Computing, IoT and Social Computing in Smart Energy Scenarios” [11]. This paper presents the use of an Edge-IoT platform and a Social Computing framework to build a system aimed at providing smart energy efficiency in a public building scenario. The proposed system was evaluated in a public building and the results make evident the notable benefits that come from applying Edge Computing to both energy efficiency scenarios and the framework itself. The main benefits of the proposal include reduced data transfer from the IoT-Edge to the Cloud and reduced Cloud, computing, and network resource costs.

The context of identification of daily life events is the context of the paper entitled “Smartphone-Based Platform for Affect Monitoring through Flexibly Managed Experience Sampling Methods” [12]. This work presents a multimodal platform which leverages the potential of smartphone sensors and the experience sampling methods to provide a continuous monitoring of the affective states and the context in a ubiquitous way. The proposed platform integrates several elements aimed at expediting real-time management of experience sampling methods questionnaires. A pilot study was conducted in order to show the potential of the proposed platform, and to evaluate its usability and its suitability for real-time assessment of affective states. The results demonstrated an excellent usability level and a good acceptance from the users and the specialists that conducted the study, and led to some suggestions for improving the data quality of mobile context-aware experience sampling methods-based systems.

The final paper of this special issue is entitled “Activity Recognition for IoT Devices Using Fuzzy Spatio-Temporal Features as Environmental Sensor Fusion” [13]. The use of spatial-temporal features by means of fuzzy logic as a general descriptor for heterogeneous sensors is proposed. This fuzzy sensor representation is highly efficient and enables devices with low computing power to develop learning and evaluation tasks in activity recognition using light and efficient classifiers. To show the methodology’s potential in real applications, an intelligent environment is presented where new Ultra-Wide-Band (UWB) location devices, inertial objects, wearable devices, and binary sensors are connected with each other to describe daily human activities. A case study developed in the UJAmI Smart Lab [14] showed the encouraging performance of the methodology in recognizing the activity of an inhabitant using efficient classifiers.
